# Development of Polyclonal Antibody against Clenbuterol for Immunoassay Application

**DOI:** 10.3390/molecules23040789

**Published:** 2018-03-29

**Authors:** Nurul Ain A. Talib, Faridah Salam, Yusran Sulaiman

**Affiliations:** 1 Functional Device Laboratory, Institute of Advance Technology, Universiti Putra Malaysia, 43400 Serdang, Malaysia; nurulaina.talib@gmail.com; 2 Department of Chemistry, Faculty of Science, Universiti Putra Malaysia, 43400 Serdang, Malaysia; 3 Biodiagnostic-Biosensor Programme, Biotechnology and Nanotechnology Research Centre, Malaysian Agricultural Research and Development Institute, 43400 Serdang, Malaysia

**Keywords:** polyclonal antibody, clenbuterol, β-agonist, antibody titer, sodium dodecyl sulfate-polyacrylamide gel electrophoresis, ELISA

## Abstract

Development of an immunoassay for clenbuterol (CLB) detection required an anti-CLB antibody as an important bioreceptor. In this study, we report our work on production and purification of a rabbit-derived polyclonal anti-CLB antibody. The antibody was then purified by nProtein A Sepharose affinity column and the antibody purity was confirmed by sodium dodecyl sulfate-polyacrylamide gel electrophoresis (SDS-PAGE) analysis. The activities of purified antibody were evaluated based on high antibody titer determined from enzyme-linked immunosorbent assay (ELISA). The sensitivity and selectivity of this antibody was evaluated and exhibits negligible cross-reactivity to antibiotics other than β-agonist families. Evaluation of the antibody as bioreceptor in immunoassay was performed using direct competitive ELISA and exhibited linear calibration plot (R^2^ = 0.9484). The antibody was used to detect the content of CLB in spiked milk samples and the recovery of more than 92% indicating significant performance as bioreceptor for the development of a rapid and simple immunoassay.

## 1. Introduction

The use of growth promoter to stimulate growth is extensively used in animal-based food product. Veterinary antibiotics including clenbuterol (CLB) from β-agonist family are illegally used as therapeutic agents [1] and usually end up as residues in the animal-based food product [2], thus impose health risks to the consumer. CLB is a legal steroid for a respiratory treatment of animal but strictly regulated for use in livestock animals. This antibiotic has anabolic and lipolytic properties in animals that cause an increase muscle tissue production and reduces fat tissue [3]. In order to produce leaner meat, this antibiotic is illegally inserted into livestock animal [4].

The use of CLB as a growth-promoting supplement was banned in the European Union [5], United States [6] and most of Asian countries [7]. However, CLB epidemic poisoning regarding consumption of beef was reported in Italy involving 15 people [8] and became a solid evidence that even though the use of CLB in the animal was banned, it is still being used uncontrollably. Meanwhile, in 2008, 68.3% from 41 analyzed milk samples tested in Turkey showed contamination of CLB, whereby the 21.7% was over the permissible level accepted by the European Union [5]. In China, CLB was found in hair analysis of hogs collected from a few farms as reported by Jia et al. [7]. Therefore, regular monitoring and screening of CLB residue in the meat-based product by regulatory agencies are very crucial to ensure the safety of consumers.

Compared with chromatographic methods such as high-performance liquid chromatography (HPLC) and liquid chromatography-mass spectrometry (LC-MS), immunoassay technology such as enzymes-linked immunosorbent assay (ELISA) and immunosensor may offer simplicity for rapid screening and monitoring purposes. The previous studies have reported on ELISA development using polyclonal and monoclonal antibody for detection of CLB in animal feeds and tissue [9,10]. However, this assay requires a high quality of antibody as bioreceptor in order to establish the immunoassay method. The immunoassay may offer high selectivity due to specific of antibody-antigen binding [11]. High-performance bioreceptors are significantly contributing to the development, sensitivity and selectivity of the immunoassay method.

The production of the polyclonal anti-CLB antibody for the use of indirect competitive ELISA was reported by He et al. [9], showing results of 25–46% cross-reactivity with salbutamol (another antibiotic from β-agonist family). Subtractive immunization as a method to produce a monoclonal anti-CLB antibody was reported by Li et al. [12]. Meanwhile, Li et al. [13] reported on the self-preparation of a monoclonal antibody specific to CLB with no cross-reaction with BSA and a kind of CLB analog. Only a small number of studies researched the production of a polyclonal anti-CLB antibody. Monoclonal antibody is often marked as superior [14] to the polyclonal antibody due to its high specificity (recognized only one epitope on an antigen) and high reproducibility (low batch-to-batch variability). Manufacturing of a low-cost antibody with high quality at high quantity is crucial for long-term advantage. Production of monoclonal antibody is relatively more expensive and requires a longer time to produce in comparison with the polyclonal antibody [15]. Polyclonal antibody is easier to store and able to handle slight variations in individual epitopes such as denaturation, polymorphism or conformational changes [16]. The use of the polyclonal antibody in immunoassay may overcome this issue. Polyclonal antibody usually has high affinity, thus able to form stable binding with larger amounts of antigen [16]. Therefore, the production of polyclonal antibody is more suitable and cost-effective. However, the produced polyclonal antibody has a high potential for cross-reactivity issue due to its ability to recognize multiple epitopes.

Herein, we are reporting on the production of high sensitivity and high selectivity of a polyclonal anti-CLB antibody towards CLB and β-agonist family with no cross-reactivity with antibiotics from other groups. Polyclonal antibody against CLB was developed by immunizing rabbits with CLB-bovine serum albumin (CLB-BSA). The rabbits were bled and the serum was extracted from the blood. Purification of the polyclonal antibody with affinity column purification step was added into the production system, instead of directly using serum to produce purer polyclonal antibody. Detection of CLB in milk samples was implemented to evaluate the performance of this antibody in real samples analysis. The objectives of this study were to produce the polyclonal antibody that, specific to CLB, has low cross-reactivity with other antibiotics and to characterize the polyclonal antibody as potential bioreceptor in the direct competitive immunoassay.

## 2. Results and Discussion

### 2.1. Polyclonal Antibody against CLB

Polyclonal antibody in this study was produced by immunizing rabbits with CLB-BSA as the immunogen. CLB is a hapten that is too small and nonimmunogenic, and is thus unable to activate the immune system by itself. The nonimmunogenic antigen can become immunogenic by conjugating the antigen to a protein carrier [17]. The use of BSA as protein carrier was a suitable strategy for raising high titer and a specific antibody to the hapten [18,19]. Therefore, by using CLB conjugated with BSA as the protein carrier, CLB-BSA has a higher potential to act as an immunogen. Immunogen is an antigen that activates the immune system to produce the immunoglobulin that is specific to the antigen.

**Figure 1:**
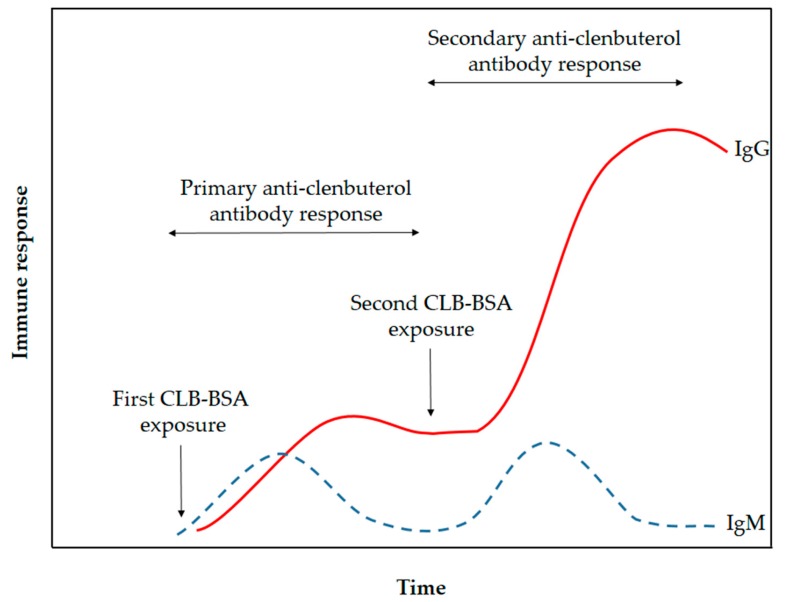
Schematic illustration of immune response towards exposure by CLB-BSA (adapted with modification from Coico et al. [22]).

The CLB-BSA solution was emulsified with Freund’s complete adjuvant (FCA) and the emulsion was injected into the rabbit. FCA was used as an immune response enhancer in primary immunization to assist in retention of CLB-BSA for a longer amount of time. FCA containing heat-killed Mycobacteria, thus attracting macrophages and initiate the long-lasting cell-mediated immune response [20]. Meanwhile, FCA was replaced with Freund’s incomplete adjuvant (FIA) for secondary and booster injections to avoid inflammation and lesions caused by the formation of granulomas. FCA and FIA contain mineral oil that acts as the vehicle to transport antigen in the lymphatic system [21]. It also causes depot effect on the antigen, thus the CLB-BSA will be retained and released slowly at the site of injection. Retention of CLB-BSA will help in the presentation of this antigen to the antigen-presenting cell, macrophages and dendritic cells, thus promoting the immune response. This will provide the continuous supply of antigen to the immune system to raise the immunoglobulin M (IgM) and immunoglobulin G (IgG). The immunoglobulin (Ig) made by the B-lymphocytes will circulate throughout the blood and lymph. After immunization, IgM level was increased first followed by IgG level as illustrated in Figure 1. The duration of the primary anti-CLB antibody response stage occurs for a month. However, the production of IgM did not increase even with continuous immunization in contrast with IgG. Repeated immunization during the secondary anti-CLB antibody response stage has increased the production of IgG. A small amount of blood was collected alternately with booster injection to provide a continuous supply of IgG. Serum was extracted from the rabbit’s blood by centrifugation to remove the red blood cell.

### 2.2. Purification of Antibody

**Figure 2:**
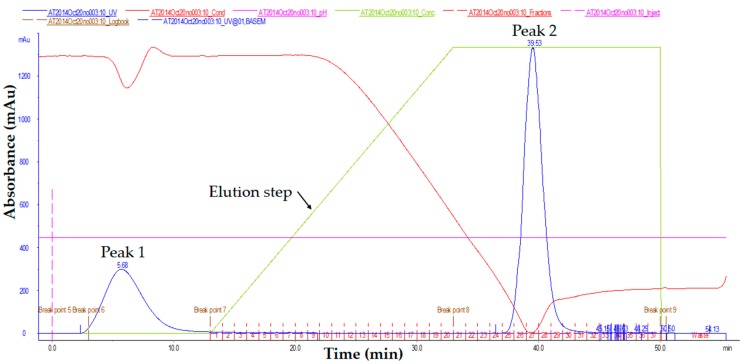
Chromatogram of IgG elution from nProtein A Sepharose affinity column using AKTAprime protein purifier system.

The serum extracted from blood can directly be used without any treatment. However, the serum contains immunoglobulins of different classes (e.g., IgG and IgM) as well as several other proteins. Therefore, in this study, the IgG from the serum was purified using affinity column chromatography purification to produce pure IgG-polyclonal antibody as illustrated in Supplementary Material Figure S1. Affinity chromatography is highly selective toward targeted protein [23], thus applicable for antibody purification in this study. Specific polyclonal antibody from serum can be isolated using nProtein A Sepharose [24] according to aseptic purification strategies. The affinity column chromatography purification activity was monitored by using built-in UV detector and the chromatogram of polyclonal antibody elution from nProtein A Sepharose is shown in Figure 2. As the serum passed through the column, the Fc (Supplementary Material Figure S2) region of IgG bonded to nProtein A through interaction with the heavy chain as shown in Supplementary Material Figure S1. Meanwhile, other proteins (such as albumin and hormones) (peak 1) were washed out during equilibrium phase. The protein A-bound IgGs were eluted out using the glycine-HCl buffer for IgG collection. The highest absorbance from peak 2 was referring to IgG, thus the isolated IgG was collected from this fraction. Due to the acid condition of the elution buffer, Tris buffer was added to neutralize the solution. In order to remove the excess salt from the previous step, the purified antibody was undergoing dialysis procedure. The IgG was freeze-dried and stored at −20 °C to maintain the stability of IgG for longer storage time.

### 2.3. Characterization of Polyclonal Antibody

#### 2.3.1. Antibody Titer Determination

**Figure 3:**
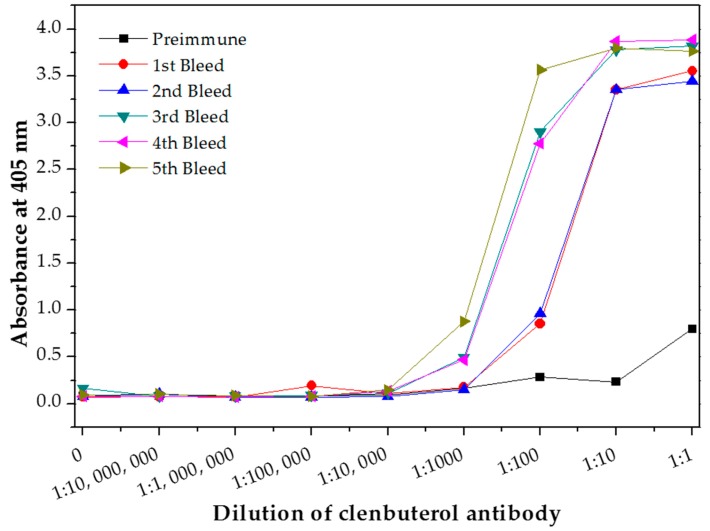
Antibody titer of the purified polyclonal anti-CLB antibody.

The antibody titer of polyclonal antibody produced in this study was determined using indirect ELISA to evaluate the antibody quality. The quantity of the antibody recognizing a specific antigen is measured by antibody titer [21]. The antibody titer is the highest dilution (lowest concentration) of antibody where the antibody reaction occurs [25]. Therefore, the quality of an antibody was evaluated based on the high titer value [24]. Sera titers of the preimmune antibody (as control experiment) and purified antibody from each bleed stages was analyzed and plotted in Figure 3. The first bleed was collected after the primary immunization, while the second, third, fourth and fifth bleed were collected after the booster immunizations. The antibody titer value is determine at the lowest dilution point where the absorbance of the bleed is overlapping with the preimmune bleed absorbance. The antibody titer value of this produced polyclonal antibody was determined as 1:1000 for the first bleed (after the primary immunization for the first CLB-BSA exposure illustrated in Figure 1) and the second bleed (after booster immunization for the second CLB-BSA exposure) meanwhile 1:10,000 for the third, fourth and fifth bleed (after booster immunization for the second CLB-BSA exposure) based on the plotted graph. Our finding is in agreement with the result reported by He et al. [9]. Highest absorbance value were obtained from purified antibody for the third, fourth and fifth bleed at concentration 1 and 0.1 mg mL^−1^. Therefore, a concentration of 1 mg mL^−1^ was chosen to be used in the direct ELISA test.

#### 2.3.2. Protein Gel Electrophoresis by Sodium Dodecyl Sulfate-Polyacrylamide Gel Electrophoresis (SDS-PAGE)

**Figure 4:**
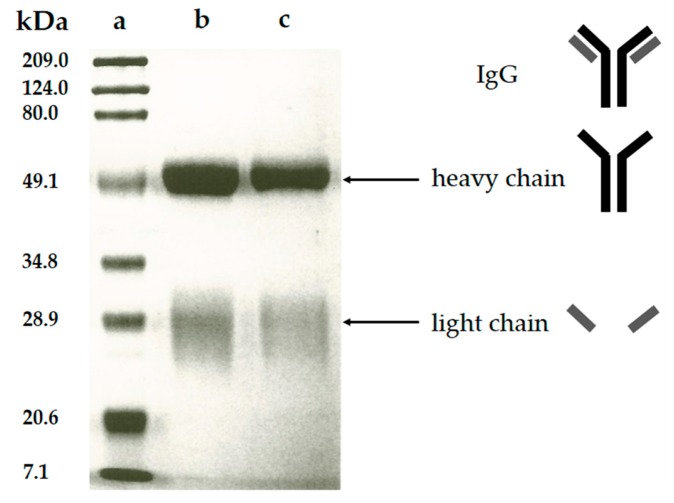
SDS-PAGE pattern of (**a**) protein marker (**b**) commercial IgG from rabbit serum (**c**) purified polyclonal antibody.

The purity of the polyclonal anti-CLB antibody produced in this study was verified by the SDS-PAGE analysis. In this analysis, the polyclonal antibody fraction migrates through the mesh-like polyacrylamide separating gel matrix towards the positively-charged cathode as the electric current passed through the electrophoresis cell. During sample preparation steps, the purified polyclonal antibody was added with β-mercaptoethanol that acted as a reducing agent [26] and underwent a heating treatment, thus breaking the polyclonal antibody structure. This denatured polyclonal antibody was forming two fractions consist of the heavy chain (~50 kDa) and light chain (~30 kDa) [27]. The heavy chain contains high molecular weight, thus migrates slower through the gel during electrophoresis. Meanwhile, the light chain fraction contains a lower molecular weight thus this polyclonal antibody fraction migrating faster and farther due to less resistance from the gel matrix. During gel staining process, Coomassie blue (dye) was bound to these chains and cause the appearance of blue stains referred as blue bands. Coomassie dye was bound to polyclonal antibody fraction through Van der Waals attraction and ionic interaction between dye sulfonic acid groups and positive polyclonal antibody fraction (protein) amine groups. The analysis of SDS-PAGE from purified polyclonal antibody (Figure 4) shows two bands which are similar to the commercial IgG from rabbit serum, meanwhile, the protein marker ladder was used as the comparison for determination of the molecular weight. The band estimated at 50 kDa and 28 kDa represent the heavy chain and light chain of the produced antibody respectively. No additional band was detected indicating that no other protein exists in the purified polyclonal antibody, thus the purification of polyclonal anti-CLB antibody was successful.

#### 2.3.3. Performance of the Polyclonal Anti-CLB Antibody Activities as Bio-Receptor

**Figure 5:**
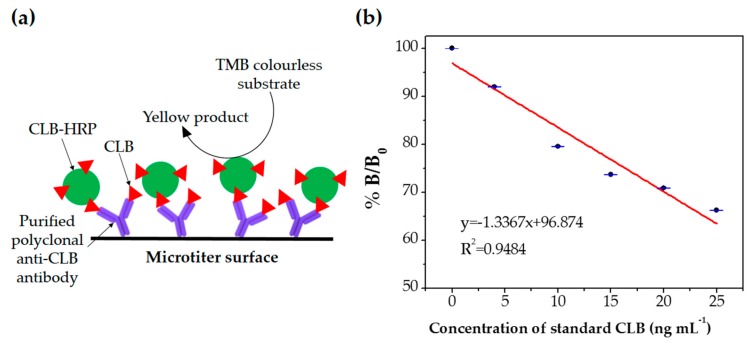
(**a**) Schematic diagram of detection principle using direct competitive ELISA; (**b**) ELISA standard CLB using direct competitive immunoassay.

Direct competitive ELISA was performed to evaluate the performance of polyclonal antibody produced as bioreceptor. Blocking agent (dry milk) was added after immobilization of the polyclonal antibody in this assay to minimize the nonspecific binding and to block the unoccupied site [28]. Different concentrations of standard CLB were tested (0, 5, 10, 15, 20 and 25 ng mL^−1^) to determine the sensitivity of the polyclonal antibody. The competition between free CLB and CLB-HRP occurs to form binding with the antibody as illustrated in Figure 5a. Samples with the low concentration of CLB contained less amount of CLB in the solution, thus more CLB-HRP were successfully bound to the antibody in the microtiter plate. Since the CLB-HRP acts as the detector and generate the color signal as the substrate TMB-H_2_O_2_ added into the system, a high signal will be produced with the low concentration of CLB thus resulting inversely proportional standard CLB plot as shown in Figure 5b. The TMB (chromogenic substrate) acts as a hydrogen donor for the reduction of hydrogen peroxide (H_2_O_2_) to water by HRP (Supplementary Material Figure S3) [29,30]. HRP acts as the peroxidase enzymes, thus produced measurable signal due to the formation of TMB/HRP/H_2_O_2_ in conjunction with redox reaction occurred [28]. Linear regression of the standard CLB plot (R^2^ = 0.9484) by ELISA and low limit of detection (LOD = 0.045 ng mL^−1^) were obtained indicating the good sensitivity of the polyclonal antibody. LOD was calculated as three times the standard deviation (σ) of zero CLB divide by the slope (3σ/slope). The ELISA assay showed that the produced polyclonal antibody had significant immunoreactivity against CLB.

#### 2.3.4. Cross-Reactivity

**Figure 6:**
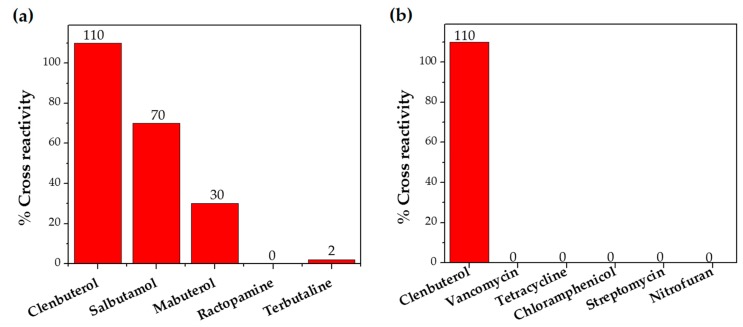
Cross-reactivity of the polyclonal anti-CLB antibody with; (**a**) antibiotics from β-agonist family and (**b**) antibiotics form other groups.

In situations when the antibody that reacts with two molecules that share epitopes, but are otherwise dissimilar in immunologic reaction, is known as cross-reactivity [22]. The specificity of this polyclonal antibody was evaluated by performing ELISA to detect other antibiotics from β-agonist families such as salbutamol, mabuterol, ractopamine and terbutaline. Antibiotics such as vancomycin, tetracycline, chloramphenicol, streptomycin and nitrofuran were also tested to evaluate the specificity of this antibody across other antibiotics groups. The cross-reactivity was calculated as 110%, 70%, 30%, 0% and 2% for clenbuterol, salbutamol, mabuterol, ractopamine and terbutaline, respectively (*n* = 3) as shown in Figure 6a. Other antibiotics from β-agonist family have similar basic structure with clenbuterol especially salbutamol (Table 1), thus a high percentage of cross-reactivity was determined. However, cross-reactivity within similar molecular structures is not an issue as defined above. Moreover, these antibiotics have similar properties and they are also categorized under banned antibiotics. These results indicate that this antibody can also be used for determinating of other β-agonist antibiotics such as salbutamol. Meanwhile, no cross-reactivity was detected for vancomycin, tetracycline, chloramphenicol, streptomycin and nitrofuran from this study (Figure 6b) implying the produced antibody does not recognize antibiotics from other than β-agonist family that are also commonly used in livestock animals. This result shows that the produced polyclonal antibody from this study is suitable to be used as bioreceptor and can specifically react to CLB even with the presence of other antibiotics in the samples. Development of highly specific immunoassay for CLB detection can be realized using this produced polyclonal anti-CLB antibody based on the performance analysis above.

#### 2.3.5. Recovery of CLB

In order to evaluate the performance of the produced antibody to capture CLB in the real sample, detection of CLB in spiked milk samples was performed. Two milk samples were spiked with 5 and 10 ng mL^−1^ standard CLB and measured after mixing with PBS (1:1). The recovery of spiked samples was calculated from calibration plot and shown in Table 2. Excellent recovery of CLB was obtained with more than 92% recovery. During the assay, the step involving blocking with dry milk was included after antibody incubation step to avoid nonspecific binding. However, there were possibilities of proteins larger than CLB (such as caseins) from the milk trapped on the well surface and blocked the antibody binding site due to steric hindrance [31,32], thus resulting in more than 100% recovery for certain samples. No complicated preparation and extraction of milk samples were performed in this analysis. The produced antibody can still recognize and capture CLB even with the existence of interference from other protein and compounds in the milk solution, implying the advantage of this produced antibody.

## 3. Materials and Methods 

### 3.1. Materials

Clenbuterol hydrochloride (CLB), Freund’s complete adjuvant (FCA), Freund’s incomplete adjuvant (FIA), glycine, 4-nitrophenyl phosphate disodium salt hexahydrate (p-NPP), 3,3′,5,5′–tetramethylbenzidine (TMB), Tween 20, tris(hydroxymethyl)aminomethane hydrochloride (Tris HCl), ammonium persulfate (APS), salbutamol, terbutaline hemisulfate salt, ractopamine hydrochloride, mabuterol hydrochloride, vancomycin, chloramphenicol, streptomycin sulfate salt, tetracycline, nitrofurantoin and glycerol were purchased from Sigma-Aldrich (St. Louis, MO, USA). Di-sodium hydrogen phosphate (Na_2_HPO_4_), sodium dihydrogen phosphate (NaH_2_PO_4_·H_2_O), sodium dodecyl sulfate (SDS), β-mercaptoethanol, acetic acid and methanol were purchased from Merck (Billerica, MA, USA). nProtein A Sepharose Fast Flow (nProtein A), non-fat dry milk, clenbuterol-bovine serum albumin (CLB-BSA), clenbuterol-horseradish peroxide (CLB-HRP), alkaline phosphate conjugated goat affinity purified antibody to rabbit immunoglobulin G (IgG-AP), 1,2-di-(dimethylamino)ethane (TEMED), clenbuterol-ovalbumin (CLB-OVA), commercial IgG from rabbit serum and ethanol was purchased from GE Healthcare (Uppsala, Sweden), Santa Cruz Biotechnology (Dallas, TX, USA), ImmuneChem (Burnaby, BC, Canada), Fitzgerald (North Acton, MA, USA), MPBiomedicals (Illkirch, France), OmniPur (Gibbstown, NJ, USA), Glory Science (Zhejiang, China) and HmBG Chemicals (Hamburg, Germany) respectively. *N*,*N*′-methylene-bis-acrylamide (bis acryl amide) 30% solution, Coomassie brilliant blue R-250 staining solution, prestained natural SDS-PAGE standards broad range (protein marker) were purchased from Bio-Rad (Hercules, CA, USA). All the solutions were prepared using deionized water (18.2 MΩ cm^−1^) from Milli-Q system (Millipore, Boston, MA, USA).

### 3.2. Preparation of Immunization Solutions

The first primary injection solution was prepared by diluting 0.45 mL of 500 µg mL^−1^ CLB-BSA and 0.75 mL of FCA in 0.75 mL of 0.01 M PBS (containing 0.01 M Na_2_HPO_4_ and 0.01 M NaH_2_PO_4_·H_2_O, pH 7.4), followed by continuous mixing until emulsion formed. The second, third and fourth primary injections were prepared by using the same method as the first primary injection solution, except that the FCA was replaced with FIA. Preparation of booster injections was the same with the primary injections using FIA.

### 3.3. Production of Polyclonal Anti-CLB Antibody

**Figure 7:**
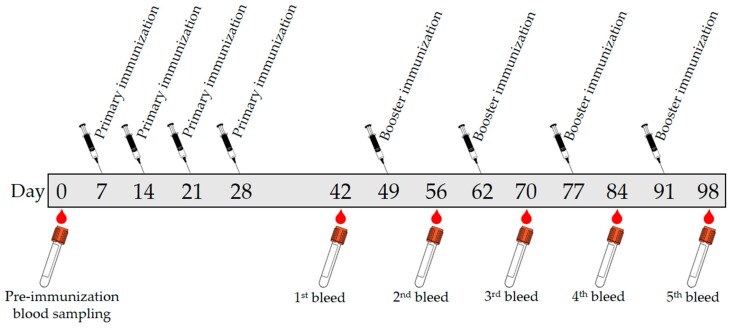
A schematic representative of immunization protocol and blood samplings.

The antibody production protocol was reviewed and approved by the Animal Ethics Committee of Malaysian Agricultural Research and Development Institute, Malaysia (Approval number 20171103/R/MAEC26; Approval date 3 November 2017). A batch of blood was collected from a rabbit (New Zealand White) by bleeding the central auricular artery on the rabbit’s ear into sterilized vacutainer tube (BD vacutainer System) and labeled as preimmune to be used as the control before the immunization started. After rested for 1 week, 1.0 mL of primary injection solution was injected subcutaneously into the rabbit. Secondary injection solutions (1.0 mL) were given after one week of rest for three weeks. Then the rabbit was given rest for two weeks before 30 mL blood was collected. A booster injection was given after two weeks. The bleeding process and booster injection were repeated at two-week interval until the fifth bleed. The immunization protocol and blood sampling were summarized in Figure 7. The blood collection was kept at room temperature for around 2 h before centrifuged for 15 min at 20 °C with 6000 rpm.

### 3.4. Purification of Antibody

The serum separated from collected blood was dissolved in distilled water (1:9) followed by slow stirring. Saturated ammonium sulfate was added drop by drop into dissolved serum (1:1) and continued by slow stirring for 30 min in the chiller at 4 °C. The mixture was transferred into 50 mL falcon tubes and centrifuged for 30 min at 15 °C with 6000 rpm. After centrifuge, the mixture was separated into supernatant and pellet. The supernatant was discarded while the pellet was dissolved in 2 mL of 0.01 M PBS (pH 7.4). The dialysis process began with the insertion of the dissolved pellet into dialysis tubing (pre-immersed with deionized water for 1 h) and secured tightly. The solution was dialysis in PBS dialysis buffer solution containing 0.01 M PBS and 0.15 M NaCl in 4 °C chiller for 4 h. After that, the dialysis buffer solution was changed. The dialysis process was continued for a total of 3 times before continued with column purification step. Column XK16/20 was packed with nProtein A Sepharose and connected to the instrument (AKTAprime). The inlet tubings from the purification system were immersed in buffer solutions accordingly. The binding buffer (0.1 M PBS, pH 7.0) and elution buffer (0.1 M glycine, pH 3.0) were degassed before used. The solution after dialysis was injected into the system for purification. The purified antibody was collected into tube collector based on chromatogram from built-in UV detector in AKTAprime software. Neutralizing buffer (1 M Tris HCl, pH 9.0) was added (100 µL/tube) into the purified antibody collected. The collection of purified antibody was undergoing dialysis with 3 times exchange of dialysis buffer solutions and proceeded to freeze dry at −80 °C.

### 3.5. Characterization of Polyclonal Antibody

#### 3.5.1. Antibody Titer ELISA

CLB-OVA with the concentration of 13 mg mL^−1^ was diluted to 100 µg mL^−1^ (in 0.01 M PBS, pH 7.4) and filled into microtiter plate with 100 µL/well before incubated overnight at 4 °C. The plate was washed three times with 250 µL/well PBST (PBS with 0.5% Tween 20). Then, blocking agent (0.05% non-fat dry milk in 0.01 M PBS, pH 7.4) was added to the microtiter plate with 250 µL/well before incubated for 1 h at 37 °C. After that, all wells were washed three times with 250 µL PBST/well. Purified antibody from each bleed was diluted to 1, 10^−1^, 10^−2^, 10^−3^, 10^−4^, 10^−5^, 10^−6^, 10^−7^ and 0 mg mL^−1^ and each dilution was inserted into the microtiter plate (100 µL/well per solution) in three replicates followed by incubation for 2 h at 37 °C. After that, all wells were washed three times with 250 µL PBST/well. Dilution of IgG-AP solution (1:1000 in 0.01 M PBS, pH 7.4) was added to the microtiter plate 100 µL/well. Incubation was done for 30 min at 37 °C, followed by washing three times with 250 µL PBST/well. Before reading the absorbance, each well was added with 100 µL of 4-nitrophenyl phosphate disodium salt hexahydrate (p-NPP) substrate and incubated for 30 min at room temperature. Then the absorbance was read at 405 nm wavelength by using ELISA reader.

#### 3.5.2. Protein Gel Electrophoresis

Preparation of sodium dodecyl sulfate-polyacrylamide gel electrophoresis (SDS-PAGE). The glass plates were cleaned with ethanol before assembling it into the gel casting mold. Solution mixture containing 4 mL of bis acryl amide, 2.5 mL of 1.5 M Tris HCl (pH 8.8), 100 µL of 10% SDS, 3.4 mL of deionized water, 50 µL of APS and 5 µL of TEMED was poured into the mold. The mixture was overlaid with deionized water and left for 20 min to form the resolving gel. The overlaid water was removed before replaced with a solution mixture of 1.7 mL of bis acryl amide, 2.5 mL of 0.5 M Tris HCl (pH 6.8), 100 µL of 10% SDS, 5.7 mL of deionized water, 50 µL of APS and 10 µL of TEMED. 8-lane comb was inserted into the mold before left for 30 min until wells were formed on the stacking gel. Then, the mold was assembled into electrophoresis unit and filled with running buffer (25 mM Tris, 192 mM glycine, 0.1% SDS). The freeze-dried purified serum and commercial IgG from rabbit serum were diluted in PBS solution and mixed with sample SDS-gel loading buffer containing 50 mM Tris HCl (pH6.8), 0.7% β-mercaptoethanol, 2% (*w/v*) SDS, 0.1% bromophenol blue and 10% (*v/v*) glycerol in ratio (9:1) before heated at 99 °C for 5 min. Diluted purified serum solution (5 µL) was loaded into the well alongside with protein marker (5 µL) and commercial IgG from rabbit serum (5 µL). The SDS-PAGE was carried out in electrophoresis cell at 120 V for 75 min. Then the gel was run until bromophenol blue reached the bottom, followed by staining the gel with staining solution. The staining solution was discarded after 20 min and replaced with destaining solution (acetic acid: methanol: deionized water in ratio 1:1:8). The gel was shaken in destaining solution for 30 min. The destaining solution was replaced with new destaining buffer every 30 min until the background became colorless.

#### 3.5.3. Direct Competitive ELISA Analysis

The purified antibody (1.0 mg mL^−1^) in this study was inserted into the microtiter plate (100 µL/well) in three replicates for each concentration of standard CLB before incubated overnight at 4 °C. All wells were then washed three times with 250 µL PBST/well. Blocking agent (0.05% non-fat dry milk in 0.01 M PBS, pH 7.4) was added to the microtiter plate with 250 µL/well before incubated for 1 h at 37 °C. Then, all wells were washed three times with 250 µL PBST/well. Standard CLB was diluted into 0, 5, 10, 15, 20 and 25 ng mL^−1^ in 0.01 M PBS (pH 7.4). Each dilution was inserted 50 µL/well per solution in three replicates along with 50 µL CLB-HRP (prepared in ratio 1:640) followed by incubation for 2 h at 37 °C. Then, all wells were washed three times with 250 µL PBST/well. Before reading the absorbance, each well was added with 50 µL/well of TMB substrate and incubated for 30 min at room temperature. Then the absorbance was read at 405 nm wavelength by using ELISA reader.

#### 3.5.4. Recovery Study

Recovery studies were performed using full cream milk from two different brands obtained from local market. The milk samples were diluted with 0.01 M PBS (pH 7.4) in ratio 1:1 [33] and vortex for 1 min. The spiked milk samples were prepared by adding an appropriate concentration of CLB diluted in 0.01 M PBS (pH 7.4), followed by vortex for 1 min. The aliquots of spiked samples (50 µL) were analyzed following direct competitive ELISA analysis as described in the previous section. Recovery of spiked CLB was calculated based on the calibration plot of standard CLB.

## 4. Conclusions

A polyclonal anti-CLB antibody was produced in rabbit and evaluated for the application as bioreceptor. A purification step using nProtein A Sepharose in affinity column chromatography has resulted in the production of the pure IgG antibody as characterized by SDS-PAGE analysis. Application of this antibody as a bioreceptor in direct-competitive ELISA has produced linear regression of standard CLB plot (R^2^ = 0.9484) with a low limit of detection (LOD = 0.045 ng mL^−1^), which indicates high sensitivity. No cross-reactivity was determined from antibiotics other than the β-agonist family, thus interference from other antibiotics was negligible. Performance of this antibody for detection of CLB in real samples analysis has resulted in more than 92% recovery, thus implementing the reliability of this produced antibody to be used as a bioreceptor for the development of CLB immunoassay. 

## Figures and Tables

**Table 1:** Antibiotic structures.

Family	Antibiotics	Structure
β-agonist	Clenbuterol	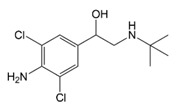
	Salbutamol	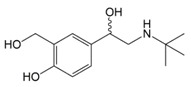
	Mabuterol	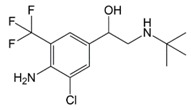
	Ractopamine	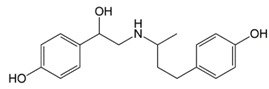
	Terbutaline	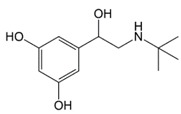
Other	Vancomycin	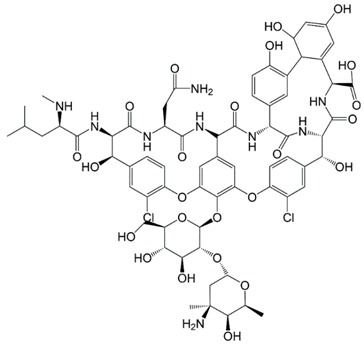
	Tetracycline	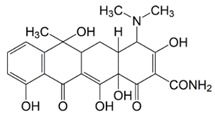
	Chloramphenicol	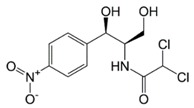
	Streptomycin	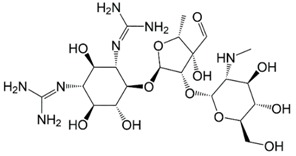
	Nitrofuran	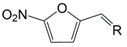

**Table 2:** Recoveries and precision of CLB in spiked milk samples (*n* = 3).

Samples	Spiked Concentration (ng mL^−1^)	Average Recovery (ng mL^−1^)	Recovery (%)	Coefficient of Variation (%)
Milk A	5	6.91	138	8.5
	10	10.17	102	10.9
Milk B	5	5.76	115	3.9
	10	9.24	92	6.9

## Supplementary Materials

Supplementary materials can be found at http://www.mdpi.com/1420-3049/23/4/789/s1. Figure S1. Illustration of IgG separation procedure in affinity column; (i) IgG specifically bound to the nProtein A; (ii) the unbound proteins were washed out; and (iii) the specific IgG was eluted out and collected. Figure S2. Schematic diagram of the IgG structure. Figure S3. Schematic of HRP-catalyzed oxidation of 3,3′,5,5′–tetramethylbenzidine.

## Abbreviations

CLB	clenbuterol
BSA	bovine serum albumin
ELISA	enzyme-linked immunosorbent assay
FCA	Freund’s complete adjuvant
FIA	Freund’s incomplete adjuvant
HPLC	high-performance liquid chromatography
HRP	Horseradish peroxidase
Ig	Immunoglobulin
LC-MS	liquid chromatography-mass spectrometry
OVA	ovalbumin
PBS	Phosphate buffer saline
p-NPP	4-nitrophenyl phosphate disodium salt hexahydrate
SDS-PAGE	sulfate-polyacrylamide gel electrophoresis
TEMED	1,2-di-(dimethylamino)ethane
TMB	3,3′,5,5′–tetramethylbenzidine
