# Supplementary material for: Development of Polyclonal Antibody against Clenbuterol for Immunoassay Application
Source: Molecules. 2018 Mar 29;23(4):789. doi: 10.3390/molecules23040789 (PMC6017646; doi:10.3390/molecules23040789)
Supplement: Supplementary file 1 [file molecules-23-00789-s001.pdf]

molecules

MDPI

Supplementary Materials

# Development of polyclonal antibody against clenbuterol for immunoassay application

**Nurul Ain A. Talib [1,2], Faridah Salam [3] and Yusran Sulaiman [1,2,*]**

[1] Functional Device Laboratory, Institute of Advance Technology, Universiti Putra Malaysia, 43400 Serdang, Selangor, Malaysia;
[2] Department of Chemistry, Faculty of Science, Universiti Putra Malaysia, 43400 Serdang, Selangor, Malaysia;
[3] Biodiagnostic-Biosensor Programme, Biotechnology and Nanotechnology Research Centre, Malaysian Agricultural Research and Development Institute, 43400 Serdang, Selangor, Malaysia;
* Correspondence: yusran@upm.edu.my; Tel.: +60-389466779; Fax: +60-389435380

**Table of Contents**


| Content | | Page no. |
|---|---|---|
| Figure S1. | Illustration of IgG separation procedure in affinity column; (i) IgG specifically bound to the nProtein A; (ii) the unbound proteins were washed out; and (iii) the specific IgG was eluted out and collected. | 3 |
| Figure S2. | Schematic diagram of the IgG structure. | 3 |
| Figure S3. | Schematic of HRP-catalyzed oxidation of 3,3′,5,5′–tetramethylbenzidine. | 6 |

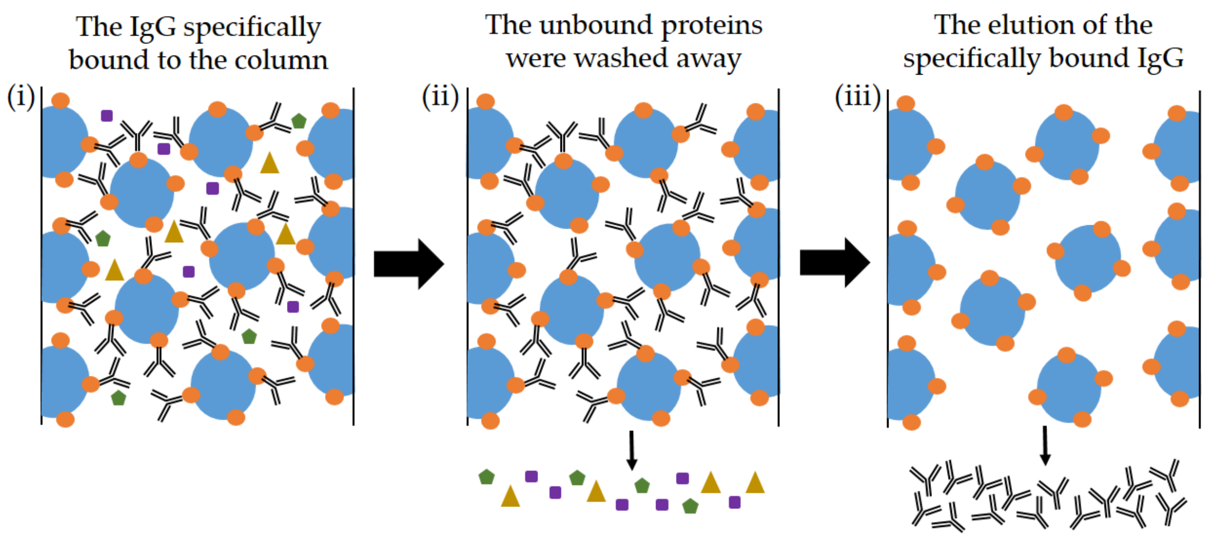


**Figure S1.** Illustration of IgG separation procedure in affinity column; (i) IgG specifically bound to the nProtein A; (ii) the unbound proteins were washed out; and (iii) the specific IgG was eluted out and collected.

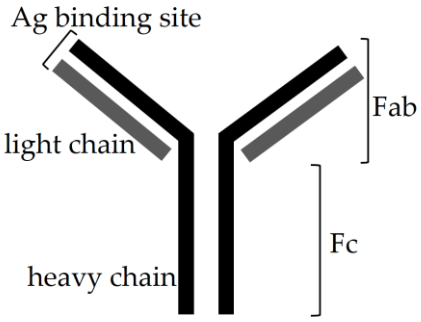


**Figure S2.** Schematic diagram of the IgG structure.

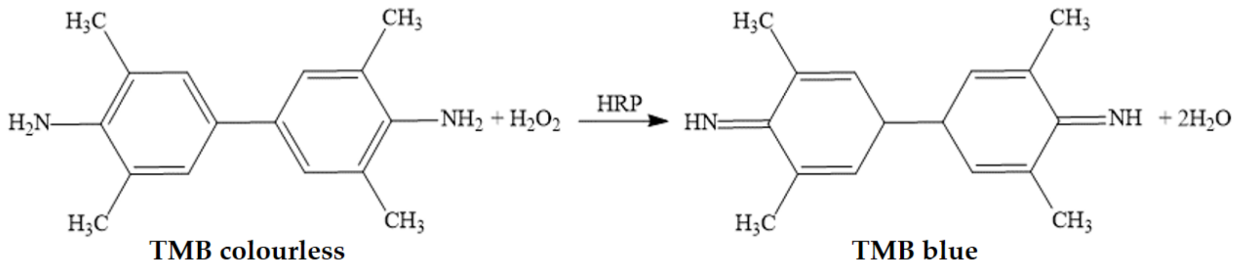


**Figure S3.** Schematic of HRP-catalyzed oxidation of 3,3′,5,5′–tetramethylbenzidine.
